# Acute Intravenous Astaxanthin Administration Modulates Hyperexcitability in Rat Nociceptive Secondary Sensory Neurons Induced by Inflammation

**DOI:** 10.3390/md24010049

**Published:** 2026-01-21

**Authors:** Risako Chida, Mamoru Takeda

**Affiliations:** Laboratory of Food and Physiological Sciences, Department of Life and Food Sciences, School of Life and Environmental Sciences, Azabu University, 1-17-71 Fuchinobe, Chuo-ku, Sagamihara 252-5201, Japan; f22001@azabu-u.ac.jp

**Keywords:** astaxanthin complementary alternative medicine, extracellular single-unit recording, spinal trigeminal nucleus caudalis, trigeminal pain, inflammation

## Abstract

Previous in vivo studies have clearly demonstrated that the intravenous administration of the carotenoid astaxanthin (AST) suppresses the excitability of rat trigeminal spinal nucleus caudalis (SpVc) neurons. This action is hypothesized to be mediated through the inhibition of both voltage-gated Ca^2+^ (Cav) channels and excitatory glutamate receptor transmission. The objective of this study was to determine whether acute intravenous administration of AST alleviates the hyperexcitability of SpVc wide dynamic range (WDR) neurons in a rat model of inflammation. Neuronal responses to both nociceptive and non-nociceptive mechanical stimulation were evaluated using an in vivo electrophysiological model. One day following inflammation induced by Complete Freund’s Adjuvant (CFA), the mechanical escape threshold was significantly reduced compared to pre-injection baseline values. Subsequently, extracellular single-unit recordings were performed on SpVc WDR neurons in anesthetized, inflamed rats. The neuronal responses to both non-noxious and noxious orofacial mechanical stimuli were then analyzed. Acute intravenous administration of AST at 1 and 5 mM elicited a dose-dependent reduction in the mean firing frequency of SpVc WDR neurons in response to noxious mechanical stimuli. This inhibition peaked within 10 min and was fully reversed after approximately 25 min. Importantly, AST preferentially inhibited the discharge frequency of SpVc WDR neurons in response to noxious stimulation, exhibiting a significantly greater effect than on the response evoked by non-noxious stimulation (41.5 ± 3.0% vs. 20.7 ± 4.2%, *p* < 0.05). Collectively, these findings demonstrate that acute intravenous administration of AST effectively suppresses noxious synaptic transmission within the SpVc during inflammation. We propose that this suppressive effect is mediated by the inhibition of upregulated Cav channels and glutamate receptors. Consequently, AST is implicated as a promising therapeutic candidate for the management of trigeminal inflammatory pain, given its potential for a favorable safety profile compared to conventional treatments.

## 1. Introduction

Nociceptive signals originating from the orofacial region are conveyed via small Aδ-fibers and unmyelinated C-fibers of trigeminal ganglion neurons to second-order neurons located within the spinal trigeminal nucleus caudalis (SpVc) [1,2,3]. Nociceptive neurons in the SpVc are functionally categorized as either nociceptive-specific (NS) or wide-dynamic range (WDR), based on their responsiveness to mechanical stimuli applied to the orofacial region, such as the facial skin. Specifically, SpVc WDR neurons respond to both noxious (painful) and non-noxious (non-painful) mechanical stimuli [2,3]. The application of graded noxious stimuli to the receptive fields results in an increased firing frequency of SpVc WDR neurons that is proportional to the stimulus intensity. This observation suggests that WDR neurons play a crucial role in encoding stimulus intensity. Rat models of orofacial inflammation induced by Complete Freund’s adjuvant (CFA) have been established to investigate the trigeminal neural pathways associated with pathological pain, where hyperexcitability of SpVc WDR neurons in response to mechanical stimuli is observed [4,5]. SpVc neurons have also been suggested to play an important role in causing hyperalgesia and/or referred pain related to dental pain [1,3,4,5,6].

Astaxanthin (AST) is a naturally occurring carotenoid that is widely distributed across a variety of living organisms [7]. Major sources include plants, microalgae, crustacean shells (e.g., crabs and shrimps), and salmon. AST demonstrates markedly greater antioxidant potency than common carotenoids, notably lutein and zeaxanthin [8,9,10]. This compound is also associated with diverse health benefits, exhibiting anti-inflammatory, anti-tumor, anti-diabetic, and immunomodulatory properties [11,12,13]. The study by Ohgami et al. [14] reported that AST dose-dependently inhibited the production of the pro-inflammatory cytokines prostaglandin E2 (PGE_2_) and tumor necrosis factor-α (TNFα) in both in vivo and in vitro models of lipopolysaccharide (LPS)-induced inflammation. Furthermore, AST suppressed the expression of cyclooxygenase-2 (Cox-2), the enzyme responsible for PGE_2_ synthesis, in primary cultures of chondrocytes and microglial cells [15,16].

We recently demonstrated that systemic administration of AST attenuates CFA-induced inflammatory mechanical hyperalgesia associated with hyperexcitability of nociceptive SpVc WDR neurons via inhibition of the Cox-2 signaling pathway [17]. Numerous studies have established that voltage-gated Ca^2+^ (Cav) channels and *N*-methyl-D-aspartate (NMDA) glutamate receptors play critical roles in peripheral and central sensitization, particularly concerning synaptic plasticity within pain transmission pathways [18,19]. In rat models of carrageenan-induced acute inflammation, significant up-regulation of Cav channel expression was observed in both dorsal root ganglion (DRG) neurons and spinal dorsal horn neurons, as previously reported by Yokoyama et al. [20]. Following the induction of colitis by trinitrobenzene sulfonic acid (TNBS) in rats, the selective up-regulation of the NMDA-NR1 receptor subunit was observed in the myenteric plexus [21]. Collectively, these data imply that inflammatory hyperalgesia is mediated, at least in part, by the enhanced expression of presynaptic Cav channels and post-synaptic NMDA glutamate receptors, leading to central sensitization. Our previous findings demonstrated that acute systemic AST administration effectively inhibits trigeminal sensory transmission and nociception in non-pathological states [22]. This short-term effect is hypothesized to stem from the inhibition of Cav channels and subsequent reduction in excitatory glutamate release. These results position AST as a promising candidate for treating trigeminal nociceptive pain with a favorable safety profile. Collectively, the evidence presented here indicates that AST, when administered intravenously, effectively suppresses the inflammation-induced hyperexcitability of nociceptive WDR neurons in the SpVc, which underlies trigeminal hyperalgesia. This protective action is achieved by targeting presynaptic Cav channels and post-synaptic NMDA receptors. Therefore, AST warrants further investigation as a potential agent for managing inflammatory hyperalgesia.

To our knowledge, no studies have currently investigated whether the acute intravenous administration of AST modulates the excitability of nociceptive neurons under inflammatory conditions. This research was designed to integrate our previous observations, namely that continuous intraperitoneal AST alleviates peripheral sensitization-driven SpVc hyperexcitation via COX-2 inhibition, while acute intravenous AST suppresses central sensitization by modulating presynaptic Cav channels and post-synaptic NMDA receptors. These experiments evaluate whether AST inhibits the inflammation-induced up-regulation of Cav and NMDA receptors. Determining the presence of short-term analgesic effects will provide insights into the clinical feasibility of AST. Therefore, the present study investigated whether intravenous AST administration, using an in vivo rat model, attenuates the CFA-induced hyperexcitability of SpVc neurons associated with hyperalgesia. This was achieved using extracellular single-unit recording techniques. Crucially, we found that the acute systemic administration of AST efficiently suppresses noxious synaptic transmission in the SpVc during inflammatory conditions. This suppression is mediated by the inhibition of up-regulated presynaptic Cav and post-synaptic glutamate receptors.

## 2. Results

### 2.1. CFA Induces Orofacial Mechanical Hyperalgesia and Edema

**Figure 1:**
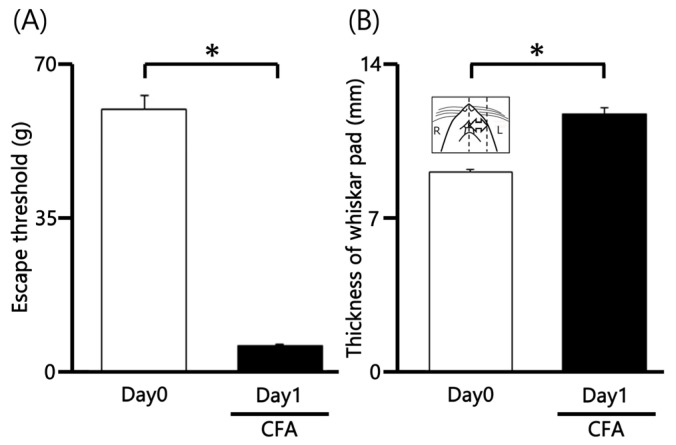
Changes in Mechanical Withdrawal Threshold and Orofacial Edema Following CFA Injection. (**A**) Mechanical withdrawal threshold (assessed via von Frey filaments) on the ipsilateral whisker pad of rats before (Day 0) and one day after (Day 1) injection of Complete Freund’s Adjuvant (CFA). The reduction in threshold indicates mechanical hyperalgesia. (**B**) Mean thickness of the ipsilateral whisker pad before and after CFA injection, demonstrating inflammatory edema. Inset: Representative region of the orofacial area used for measuring CFA-induced edema thickness. * *p* < 0.05.

We first evaluated mechanical hyperalgesia in CFA-inflamed rats by applying von Frey filaments to the orofacial skin (whisker pad). CFA injection significantly reduced the mechanical withdrawal threshold on the ipsilateral whisker pad area from 60.3 ± 2.9 g in non-injected rats to 6.2 ± 0.3 g at Day 1 post-injection (*n* = 9, *p* < 0.05; Figure 1A). In contrast, the mechanical withdrawal threshold on the contralateral whisker pad area remained unchanged after CFA injection (62.1 ± 4.5 g vs. 61.3 ± 2.3 g, *n* = 9, not significant). Furthermore, the mean thickness of the ipsilateral whisker pad was also significantly increased following CFA administration (9.1 ± 0.1 mm vs. 11.8 ± 0.3 mm, *n* = 9, *p* < 0.05; Figure 1B), indicating localized edema.

### 2.2. General Properties of SpVc Neurons Innervating Inflamed Facial Skin

**Figure 2:**
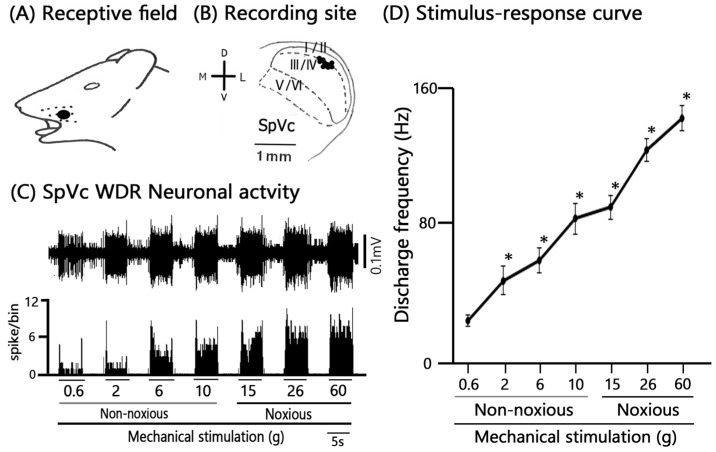
General Characteristics of SpVc Wide Dynamic Range (WDR) Neurons Innervating Orofacial Skin. (**A**) Receptive field (RF) location of recorded SpVc neurons on the ipsilateral whisker pad of the facial skin (Blacken area). (**B**) Distribution map of the recording sites (black circles) for SpVc WDR neurons (*n* = 9) that responded to both non-noxious and noxious mechanical stimulation applied to the facial skin. (**C**) Representative example trace showing the firing patterns of a single SpVc WDR neuron induced by non-noxious and noxious mechanical stimulation. (**D**) Stimulus-response curve for the recorded SpVc WDR neurons (*n* = 9). The curve illustrates the firing frequency increasing proportionally to the stimulus intensity.* *p* < 0.05 for comparison of 0.6 g vs. 2 g, 6 g, 10 g, 15 g, 26 g, and 60 g.

We performed extracellular single-unit recordings from nine SpVc neurons in Day 1 CFA-inflamed rats. Subsequent pharmacological testing with intravenous AST (1 mM, *n* = 3; 5 mM, *n* = 6) was conducted on all nine of these SpVc units. Figure 2A shows a typical example of an inflamed receptive field of TG neurons responding to non-noxious and noxious mechanical stimulation in the whisker pad. Single-unit recording sites are shown in Figure 2B and were mainly distributed in maxillary branches in the SpVc [17,23]. There were no obvious differences in the location of the recording sites (Figure 2B). All nine recorded neurons (*n* = 9, 100%) were categorized as WDR neurons, characterized by a firing frequency that increased proportionally to the intensity of graded mechanical stimulation applied to the most sensitive area of the RF (Figure 2C,D). The mean threshold for mechanical stimulation-induced spiking was 0.5 ± 1.2 g. Furthermore, 100% (*n* = 9) of the recorded units exhibited spontaneous discharges at a frequency of 1.7 ± 0.6 Hz, which is consistent with our prior findings.

### 2.3. SpVc Neuronal Activity After CFA Treatment in Response to Noxious and Non-Noxious Stimuli Following AST Administration

**Figure 3:**
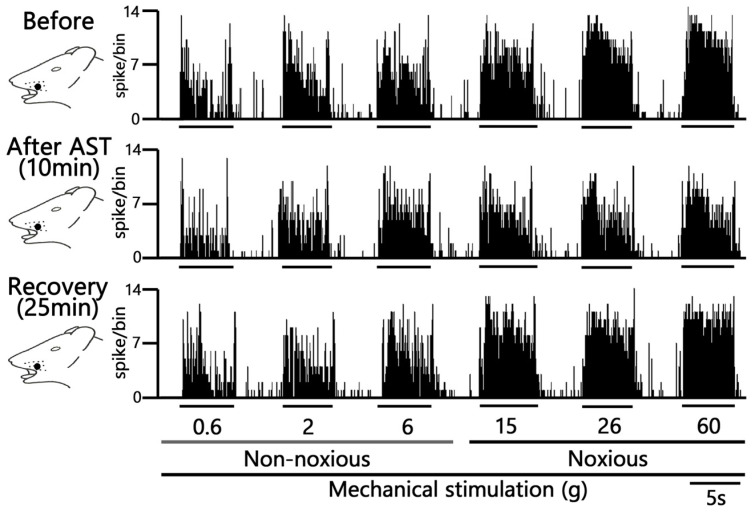
Acute Effects of Intravenous Astaxanthin (AST) on SpVc WDR Neuronal Firing. Typical examples of SpVc WDR neuronal activity evoked by non-noxious (0.6, 2 and 6 g), noxious (15, 26 and 60 g) mechanical stimuli and noxious pinch mechanical stimulation: before and 10 min and 25 min after i.v. administration of 5 mM AST.

A typical example trace demonstrating the inhibitory effect of AST (5 mM) in the example figure on neuronal activity evoked by non-noxious mechanical stimulation is presented in Figure 3. Specifically, 5 mM i.v. AST administration resulted in the inhibition of neuronal firing evoked by non-noxious mechanical stimuli (0.6–10 g), with the peak effect observed approximately 10 min post-injection. The inhibitory effect was transient, with neuronal activity recovering to pre-injection control levels within approximately 25 min. Crucially, AST administration did not induce any significant changes in either the mechanical threshold for spiking or the receptive field size. Although there was an observed tendency for a decrease in the spontaneous discharge rate of the neurons (Before vs. after, 1.7 ± 0.6 vs. 1.2 ± 0.4 Hz, this change was not statistically significant.

**Figure 4:**
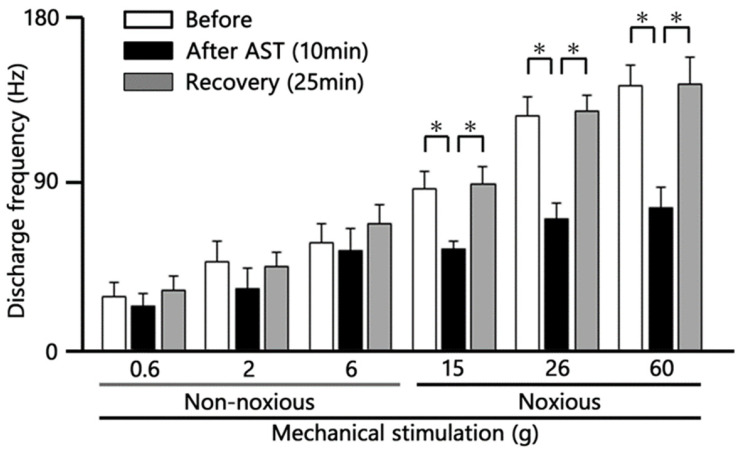
Time Course Analysis of Intravenous Astaxanthin (AST) Effects on SpVc WDR Neuron Firing. Quantification of the mean firing frequency of Spinal Trigeminal Nucleus Caudalis (SpVc) Wide Dynamic Range (WDR) neurons following intravenous (i.v.) AST administration (5 mM dose). * *p* < 0.05 before vs. 10 min after AST; * *p* < 0.05, 10 min after AST vs. recovery (25 min) (*n* = 6).

We further quantified the effect of intravenous (i.v.) AST on the firing rate of SpVc neurons following both non-noxious and noxious mechanical stimulation. The inhibitory effect of AST on neuronal activity evoked by non-noxious mechanical stimulation (0.6–10 g) was quantified and summarized in Figure 4. Although there was an observed tendency for the mean firing rates to decrease after AST injection compared to pre-injection controls, this change was not statistically significant (*n* = 6). This transient decrease tended to recover to control levels within approximately 25 min.

Representative examples illustrating the inhibitory effect of 5 mM i.v. AST on the excitability of SpVc neurons evoked by noxious stimulation (15, 26, and 60 g) are shown in Figure 3. The mean firing rates evoked by noxious stimuli (15, 26, and 60 g) decreased significantly after AST injection compared with control levels (*p* < 0.05, *n* = 6; Figure 4). The inhibitory effect was transient, with the neuronal activity (specifically at 60 g) returning to control levels within approximately 25 min (*p* < 0.05, *n* = 6).

**Figure 5:**
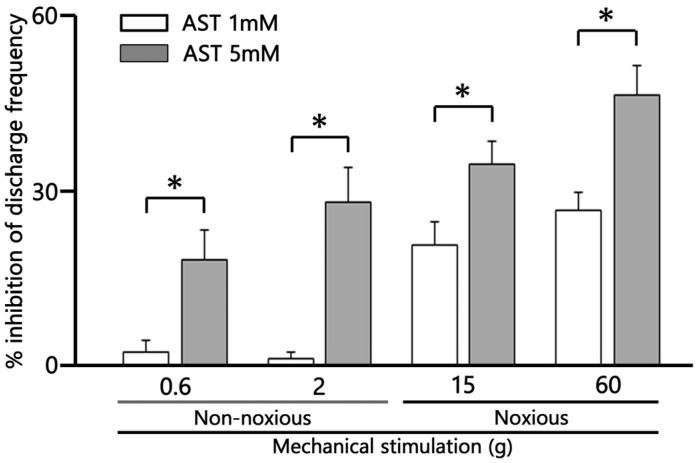
Dose-dependent suppression by astaxanthin (AST) of the mean firing frequency of trigeminal spinal nucleus caudalis (SpVc) wide dynamic range (WDR) neurons responding to non-noxious, noxious, and noxious mechanical stimulation. * *p* < 0.05, 1 mM (*n* = 3) vs. 5 mM AST (*n* = 6), i.v.

AST exhibited significant dose-dependent (1–5 mM) suppression of non-noxious mechanical stimulation-evoked SpVc WDR neuron firing (Figure 5; 1 mM vs. 5 mM, *p* < 0.05). AST exhibited significant dose-dependent (1–5 mM) suppression of noxious mechanical stimulation-evoked SpVc WDR neuron firing (Figure 5; 1 mM vs. 5 mM, respectively, *p* < 0.05).

### 2.4. SpVc WDR Neuronal Activity in Response to Noxious vs. Non-Noxious Stimuli After AST

**Figure 6:**
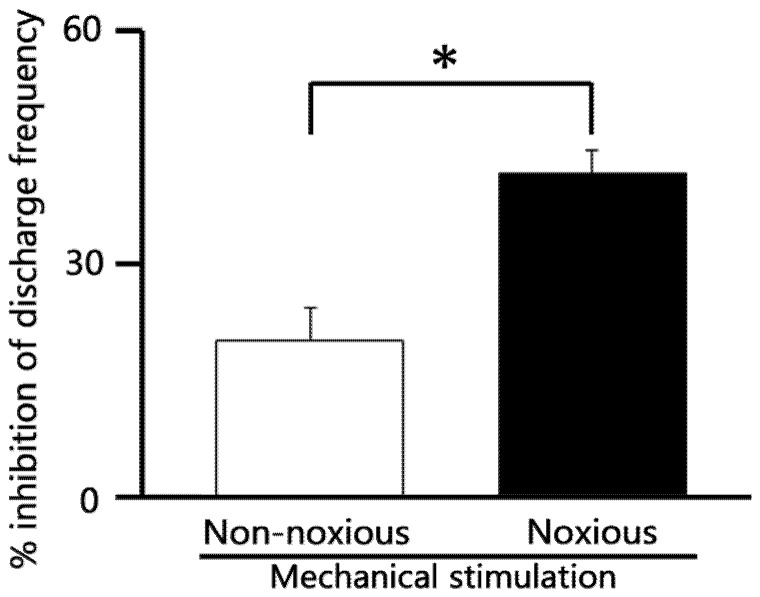
Comparison of 5 mM astaxanthin (AST) -induced inhibition of SpVc WDR neuronal discharge frequency between non-noxious and noxious stimulation. * *p* < 0.05, non-noxious vs. noxious stimulation. (*n* = 6).

To compare the relative efficacy of AST on different pain modalities, we assessed the inhibitory effect of 5 mM intravenous (i.v.) AST on neuronal responses to both non-noxious and noxious stimuli. As summarized in Figure 6, the relative magnitude of inhibition exerted by AST on SpVc WDR neuronal discharge frequency was significantly greater for noxious mechanical stimulation compared to non-noxious mechanical stimulation (*p* < 0.05). This finding suggests that AST possesses a selective inhibitory action, targeting the transmission of noxious (painful) signals more effectively than non-noxious signals within the trigeminal pain pathway.

## 3. Discussion

### 3.1. Acute Intravenous Administration of AST Attenuates Hyperexcitability of Inflamed SpVc WDR Neurons

The present study investigated whether the acute intravenous administration of AST could attenuate the inflammation-induced hyperexcitability of spinal SpVc WDR neurons.

From this study, we report the following main findings: (i) Mechanical hyperalgesia was confirmed by a significantly lower mean mechanical escape threshold in CFA-inflamed rats on Day 1 compared to the pre-CFA baseline; (ii) The mean thickness of the edematous area in the whisker pad was significantly increased following CFA injection; (iii) Consistent with our previous studies [17,23], CFA-induced inflammation resulted in neuronal hyperexcitability, characterized by an augmented decrease in mechanical threshold, and increased spontaneous and evoked discharges of SpVc WDR neurons; (iv) In Day 1 inflamed rats, AST significantly inhibited the mean firing frequency of SpVc neurons in a dose-dependent manner in response to both non-noxious and noxious mechanical stimuli. The maximal inhibitory effect on discharge frequency was observed within 15 min, lasted for 25 min, and was reversible; (v) AST preferentially inhibited the discharge frequency of SpVc WDR neurons in response to noxious stimulation, demonstrating a significantly greater effect compared to its action on non-noxious responses; and (vi) vehicle administration showed no effect on the discharge frequency of SpVc WDR neurons in response to either non-noxious or noxious mechanical stimuli. Furthermore, comparison with data from our previous studies [22] revealed no significant difference in the percentage inhibition of noxious stimulation-induced discharge by 5 mM AST between naïve and CFA-inflamed rats (naïve vs. inflamed; 40.3 ± 11.5 vs. 41.5 ± 2.9 Hz). Collectively, these findings demonstrate that the intravenous injection of the carotenoid, AST, effectively suppresses the excitability of secondary SpVc WDR sensory neurons.

### 3.2. The Hyperexitability of SpVc WDR Neurons Is Suppressed by AST via a Mechanism Involving the Central System Under Inflammatory Conditions

Nociceptive signaling is generally understood to involve four main steps [6,24]: (1) initial transduction of external stimuli at peripheral terminals; (2) subsequent generation of action potentials; (3) propagation of these potentials along axons; and (4) final transmission at central terminals, which serve as the presynaptic elements of the initial synapses within the central nervous system sensory pathways.

Previous research has demonstrated that the dietary carotenoid, lutein, can mitigate inflammation-induced mechanical hyperalgesia by suppressing SpVc WDR hyperactivity via peripheral Cox-2 signaling [25]. Similarly, we have recently reported that the systemic administration of AST attenuates CFA-induced inflammatory mechanical hyperalgesia associated with hyperexcitability of nociceptive SpVc WDR neurons, potentially through the inhibition of the Cox-2 signaling pathway [17]. However, no study has yet determined whether the acute intravenous administration of AST in vivo attenuates CFA-induced inflammatory hyperexcitability of SpVc neurons associated with hyperalgesia in rats, specifically utilizing extracellular single-unit recording to assess neuronal activity.

Following peripheral inflammation and/or nerve injury, inflammatory mediators such as PGE2 bind to G-protein-coupled E-type prostanoid receptors (EPs). This binding induces the activation of protein kinases A (PKA) and C (PKC) in nociceptive peripheral terminals, which subsequently leads to the phosphorylation of mechanosensitive sodium and potassium ion channels and receptors [4,6]. Consequently, the activation threshold for transducer channels, such as transient receptor potential ankyrin 1 (TRPA1) and acid-sensing ion channel 3 (ASIC3), is reduced. This leads to increased membrane excitability of the peripheral terminals and the subsequent conduction of a higher frequency of action potentials toward the presynaptic central terminals of the SpVc [4,6]. This cascade promotes the massive release of glutamate into the synaptic cleft. Glutamate subsequently binds to up-regulated post-synaptic glutamate receptors, thereby augmenting excitatory post-synaptic potentials (EPSPs). This culminates in a barrage of action potentials being transmitted to the higher centers of the pain pathways, establishing a state of heightened sensitivity known as peripheral sensitization [4,6].

Cav channels are classified into two main groups: low-voltage-activated (LVA) channels (T-type) and high-voltage-activated (HVA) channels (L, P/Q, N, and R types) [26]. Both N-type and T-type Cav channels significantly mediate the primary afferent nociceptive signaling pathway. Specifically, HVA N-type channels are predominantly localized in the presynaptic regions of laminae I and II within the dorsal horn [26,27,28]. Dolphin [19] previously reported that Cav channels play a pivotal role in converting electrical impulses into the exocytotic release of neurotransmitters. These channels act as the primary conduits for Ca^2+^ ions, which trigger the fusion of neurotransmitter-containing vesicles with the presynaptic membrane. Furthermore, Yokoyama et al. [18] reported the significant up-regulation of N-type Cav channel expression in both DRG neurons and spinal dorsal horn neurons in a carrageenan-induced acute inflammation rat model. This evidence suggests that up-regulated Cav channels may be a molecular target for AST-induced analgesia under inflammatory conditions. Action potentials propagating along DRG neurons (C- and Aδ-afferents) open these presynaptic N-type Cav channels, which subsequently triggers the release of nociceptive transmitters—such as glutamate, substance P, and calcitonin-gene-related peptide (CGRP)—onto spinal interneurons and projection neurons [26]. A previous study demonstrated that AST dose-dependently inhibits glutamate release from rat cortical synaptosomes by suppressing presynaptic Cav and the MAPK signaling cascade [29]. That study further showed that the effect of AST on triggered glutamate release was abolished by N-, P-, and Q-type Cav channel blockers [29]. Taken together with the results of the present study, we speculate that the systemic administration of AST suppresses trigeminal nociceptive neuronal excitability, likely acting via up-regulated N-type Cav channels, putatively at the presynaptic terminal of trigeminal ganglion (primary) neurons.

T-type Cav channels are known to be dominantly expressed in small- and medium-diameter sensory neurons, which correspond roughly to unmyelinated C- and myelinated Aδ-afferent neurons, respectively [30,31,32,33]. Todorovic and Todorovic [33] observed that an increase in the amplitude of T-type Cav currents reduced the neuronal excitability threshold, thereby increasing the probability of burst-firing. Furthermore, the up-regulation of T-type Cav channels has been reported under inflammatory conditions [34]. Given that pharmacological blockade of T-type Cav channels by Z944 in the trigeminal system induces analgesia following nerve injury, Gambeta et al. [32] demonstrated that these channels are key regulators of neuronal activity and play a pivotal role in trigeminal pain. It is therefore plausible that AST may inhibit SpVc neuronal hyperexcitability by suppressing these up-regulated T-type Cav channels. However, further investigation is necessary to elucidate this possibility.

Woolf and Salter [18] reported that NMDA glutamate receptors play a critical role in central sensitization, particularly by enhancing synaptic plasticity—a process that increases pain signaling gain and contributes to the pathogenesis of pain. A recent study demonstrated that AST helps alleviate neuropathic pain by inhibiting the activity of the NMDA glutamate receptor, particularly the subtype NR2B protein, which is crucially involved in nociception [35]. The NR2B subunit is a key tyrosine-phosphorylated protein; its phosphorylation is proposed to increase Ca^2+^ entry through the receptor, a process critical for both central sensitization and NMDA-dependent synaptic plasticity [36,37]. Moreover, the selective up-regulation of the NMDA-NR1 receptor subunit has been observed in the myenteric plexus following TNBS-induced colitis in rats [21]. Collectively, these findings suggest that the up-regulation of post-synaptic glutamate NMDA receptors contributes significantly to inflammatory hyperalgesia.

In the present study, we observed that AST significantly inhibited the mean firing frequency of SpVc neurons in response to noxious mechanical stimuli in a dose-dependent and reversible manner. Crucially, AST preferentially inhibited the discharge frequency of SpVc WDR neurons in response to noxious stimulation, showing a significantly greater effect than on the response to non-noxious stimulation. Although the precise mechanism underlying the differential sensitivity of AST to nociceptive versus non-nociceptive inputs remains elusive, it is postulated that AST predominantly inhibits central sensitization. This occurs through two primary pathways: the suppression of excitatory synaptic transmission—including inflammation-dependent up-regulation of Cav and NMDA channels in the SpVc—and the enhancement of descending inhibitory systems, such as endogenous opioidergic mechanisms, under inflammatory conditions. However, further investigation is necessary to elucidate this possibility.

### 3.3. Functional Significance of the Suppressive Effect of AST on the Hyperexcitability of SpVc Neurons

Complementary and Alternative Medicine (CAM) is typically defined by current Western medicine as a medical system that lacks sufficient scientific validation and clinical application. CAM generally encompasses modalities such as herbal medicines and acupuncture. Recently, CAM has been increasingly utilized by patients whose symptoms are refractory to conventional Western medical treatments, such as pharmacotherapy. Consequently, CAM is expected to hold significant promise in the management of chronic pain [38,39,40]. Furthermore, previous studies have reported that various dietary constituents can potentially modulate protective biological mechanisms, particularly those involved in the cardiovascular, neural, and anticancer systems [41,42].

AST is a naturally occurring carotenoid widely distributed in various organisms, including plants, microalgae, crustacean shells, and salmon [7]. AST has been demonstrated to possess numerous biological activities, such as anti-inflammatory, anti-tumor, anti-diabetic, and immunomodulatory effects [11,12,13,43]. Recently, we reported that the systemic administration of AST attenuates inflammatory mechanical hyperalgesia associated with the hyperexcitability of nociceptive SpVc WDR neurons, likely through the inhibition of the Cox-2 signaling pathway [17]. These findings support the proposal that AST holds potential as a therapeutic agent within CAM strategies to prevent or manage trigeminal inflammatory mechanical hyperalgesia, potentially serving as an alternative to non-steroidal anti-inflammatory drugs (NSAIDs) [17].

The present study demonstrated that acute intravenous administration of AST effectively suppresses noxious synaptic transmission within the SpVc during inflammation. We propose that this suppressive effect is mediated presumably by the inhibition of up-regulated Cav channels and glutamate receptors. This finding implicates AST as a promising therapeutic candidate for the management of trigeminal inflammatory pain, potentially offering a favorable safety profile compared to conventional treatments. This mechanism is supported by evidence that N- and P/Q-type Cav channel blockers exhibit analgesic effects in various pain models, as demonstrated through behavioral and in vivo electrophysiological experiments [44]. For instance, spinal administration of ω-conotoxin GVIA (an N-type Cav channel blocker) reduced spinal neuronal responses to both noxious and non-noxious pressure applied to the inflamed knee in anesthetized rats with acute inflammatory pain [45].

Alternatively, given the established role of glutamate receptor antagonists in the management of migraine [46] and the evidence that systemic AST administration suppresses excitatory synaptic transmission, including NMDA receptor activity, it is plausible to hypothesize that AST might alleviate primary headache syndromes, such as migraine and cluster headache. In other words, AST may exert an effect equivalent to that of the NMDA receptor blocker ketamine, which is used as an intravenous anesthetic. This hypothesis is further substantiated by evidence that AST administration ameliorated neuropathic pain by antagonizing the effect of NMDA glutamate receptors [35]. Previously, using a multi-barrel electrode, we demonstrated that the iontophoretic application of L-glutamate induced the mean firing frequency of an SpVc WDR neuron responding to noxious mechanical stimulation, and this response was inhibited by the intravenous administration of polyphenol, resveratrol [47]. Therefore, further studies are warranted to confirm whether AST modulates the glutamate-induced SpVc WDR neuronal discharge using a multi-barrel electrode setup.

## 4. Materials and Methods

Ethical approval for all experiments was granted by the Animal Use and Care Committee of Azabu University (Authorization No. 230120-11). All procedures adhered strictly to the ethical rules established by the International Association for the Study of Pain [48]. Conscientious efforts were maintained throughout the study to reduce the number of animals utilized and to ensure the alleviation of any potential suffering.

### 4.1. Animal Preparation and Induction of Inflammation

Adult male Wistar rats (weighing 225–275 g) were maintained on a fixed 12 h light/dark cycle (lights on 07:00–19:00) with the ambient temperature controlled at 24 °C ± 1 °C. Food and water access was ad libitum. Given the reported, yet not fully understood, sex differences in responses to experimental pain [49], this study utilized only male rats. Electrophysiological recordings were conducted in 9 animals. Anesthesia was induced in each animal with 3–5% isoflurane, followed by the injection of complete Freund’s adjuvant (CFA) (0.05 mL of a 1:1 oil/saline suspension) into the left facial skin, as previously detailed. Behavioral experiments were performed prior to and 1 day following the CFA injection.

### 4.2. Mechanical Threshold for Escape Behavior

Mechanical sensitivity was determined by assessing the escape threshold, as described by previous studies [17]. Starting 1 day post-CFA injection, mechanical hyperalgesia was quantified in both the ipsilateral and contralateral facial skin areas utilizing a set of Semmes-Weinstein Monofilaments (von Frey hairs; North Coast Medical, Morgan Hill, CA, USA). Stimuli ranging from 0.06 g to 100 g were applied to the whisker pad in an ascending order, with each force applied in triplicate. The mechanical escape threshold was established as the lowest stimulus intensity that elicited a head withdrawal response in at least one of the three applications. This determination followed the established protocol [17,21]. To investigate the anti-inflammatory effect of AST, CFA-induced edema of the whisker pad was monitored. Edema was evaluated by measuring the thickness of the whisker pad region before and after the CFA injection (Figure 1B, inset), as per established procedures [17].

### 4.3. Extracellular Single-Unit Recording of SpVc WDR Neuronal Activity

One day post-CFA or vehicle injection, electrophysiological recordings were performed on 9 adult male Wistar rats, following established protocols.

#### 4.3.1. Animal Preparation and Anesthesia

Rats were initially sedated with 3% isoflurane. Anesthesia was maintained via a jugular vein cannula delivering a mixture of medetomidine (0.3 mg/kg), midazolam (4.0 mg/kg), and butorphanol (5.0 mg/kg), supplemented as required (0.25–0.45 mL/kg/h). An adequate anesthetic plane was confirmed by the absence of a paw pinch response. Rectal temperature was maintained at 37.0 °C ± 0.5 °C using a homeothermic blanket (FHC Aspen, Tokyo, Japan). A 2% lidocaine solution (Xylocaine) was consistently applied to all wound margins for local analgesia throughout the procedure.

#### 4.3.2. Surgical Access

Animals were secured in a stereotaxic frame (SR-50; Narishige, Tokyo, Japan). The neck muscles were separated at the midline, and the medullary brainstem was exposed by careful incision of the atlanto-occipital ligament and dura mater.

#### 4.3.3. Unit Recording

Extracellular single-unit activity was recorded from the ipsilateral spinal trigeminal subnucleus caudalis (SpVc) using a tungsten microelectrode (3–5 MΩ). Microelectrode positioning was guided by the stereotaxic coordinates of Paxinos and Watson [47], adjusted in 10 μm increments using a micromanipulator (SM-11 and MO-10; Narishige, Tokyo, Japan). The neural signal was amplified (DAM80; WPI, Sarasota, FL, USA), filtered (0.3–10 kHz), monitored (SS-7672; Iwatsu, Tokyo, Japan), and subsequently recorded for analysis using a Power Lab system with Chart ver. 5 software (AD Instruments, Oxford, UK) [17,21].

### 4.4. Experimental Protocols

#### 4.4.1. Extracellular Recording of SpVc WDR Neurons

We recorded extracellular single-unit WDR responses in the SpVc to mechanical stimulation of the whisker pad. To prevent peripheral mechanoreceptor sensitization, the receptive field (RF) general location on the left whisker pad was first rapidly located using a paintbrush. Neurons were characterized as Wide Dynamic Range (WDR) units if they exhibited a graded response to a series of von Frey hairs, including both non-noxious (0.2–10 g) and noxious (15–60 g) mechanical stimuli. Each stimulus was applied for 5 s, separated by 5 s intervals [17,23].

#### 4.4.2. Neuronal Characterization and Mapping

Once nociceptive SpVc WDR neurons were identified, their mechanical stimulation threshold was determined, and the RF size was documented. RF boundaries were mapped by applying von Frey hairs to the facial skin and tracing the responsive area onto a life-sized rat diagram [17,23].

#### 4.4.3. Data Analysis

WDR neuronal activity upon mechanical stimulation was quantified by subtracting the spontaneous discharge frequency (assessed over 2–5 min) from the stimulus-evoked activity. Peristimulus histograms (100 ms bins) were constructed for each stimulus. Average spontaneous and mechanically induced discharge frequencies, along with average mechanical thresholds, were calculated.

#### 4.4.4. Rationale for Focusing on WDR Neurons

Given that SpVc WDR neurons are crucially involved in mechanical hyperalgesia [4,6], and evidence suggests nociceptive specific (NS) neurons may convert to WDR neurons following CFA-induced inflammation [1,3], this study focused exclusively on the impact of AST on nociceptive SpVc WDR neuronal activity, thus omitting the investigation of NS neurons.

#### 4.4.5. Recording Site Localization

The recording locations within the SpVc were identified relative to the obex, medial line, and the surface of the medullary dorsal horn using micromanipulator readings, consistent with the rat brain atlas [50] and our prior studies [17,21].

### 4.5. Data Analysis

All values are expressed as the mean ± standard error of the mean (SEM). Statistical analysis was performed using a one-way repeated measures ANOVA. Subsequent comparisons for the behavioral results were conducted using the Tukey–Kramer post hoc test (Excel Statcel 4). A *p*-value of < 0.05 was considered to indicate statistical significance.

## 5. Conclusions

The present study provides evidence that acute intravenous administration of AST effectively suppresses noxious synaptic transmission within the SpVc during inflammation. We propose that this suppressive effect is mediated possibly by the inhibition of up-regulated Cav channels and glutamate receptors. This finding implicates AST as a promising therapeutic candidate for the management of trigeminal inflammatory pain, potentially offering a favorable safety profile compared to conventional treatments. To verify the proposed hypothesis that acute systemic AST alleviates inflammation-induced hyperexcitability of SpVc WDR neurons, future pharmacological investigations must focus on two key areas: (i) identifying the specific Cav channel subtypes involved in AST-induced suppression using selective inhibitors and (ii) clarifying the potential involvement of NMDA receptor signaling in these inhibitory actions.

## Data Availability

All data from this study are included in the main body of the article.
